# Isolation of microplastics in biota-rich seawater samples and marine organisms

**DOI:** 10.1038/srep04528

**Published:** 2014-03-31

**Authors:** Matthew Cole, Hannah Webb, Pennie K. Lindeque, Elaine S. Fileman, Claudia Halsband, Tamara S. Galloway

**Affiliations:** 1 Plankton Group, Plymouth Marine Laboratory, Prospect Place, The Hoe, Plymouth PL1 3DH, UK; 2 College of Life and Environmental Sciences: Biosciences, Geoffrey Pope Building, University of Exeter, Stocker Road, Exeter EX4 4QD, UK; 3 School of Geography, Earth and Environmental Science, Plymouth University, Drake Circus, Plymouth PL4 8AA, UK; 4 Akvaplan-niva AS, FRAM – High North Research Centre for Climate and the Environment, N-9296 Tromsø, Norway

## Abstract

Microplastic litter is a pervasive pollutant present in aquatic systems across the globe. A range of marine organisms have the capacity to ingest microplastics, resulting in adverse health effects. Developing methods to accurately quantify microplastics in productive marine waters, and those internalized by marine organisms, is of growing importance. Here we investigate the efficacy of using acid, alkaline and enzymatic digestion techniques in mineralizing biological material from marine surface trawls to reveal any microplastics present. Our optimized enzymatic protocol can digest >97% (by weight) of the material present in plankton-rich seawater samples without destroying any microplastic debris present. In applying the method to replicate marine samples from the western English Channel, we identified 0.27 microplastics m^−3^. The protocol was further used to extract microplastics ingested by marine zooplankton under laboratory conditions. Our findings illustrate that enzymatic digestion can aid the detection of microplastic debris within seawater samples and marine biota.

Marine debris encompasses a range of anthropogenic material including glass, wood, metals, fabrics and plastic^1^. As rates of plastic production are rising rapidly, and owing to the fact that plastic can take decades, if not centuries, to fully degrade, we consider that plastic litter is an ever increasing environmental issue^2^,^3^. Of particular concern are microplastics: plastic fragments, fibres and beads <5 mm in diameter, manufactured to be microscopic in size, or derived from the degradation of larger plastic debris^4^. This microplastic litter has been identified in marine habitats across the globe^5^, and more recently has been sampled in freshwater systems^6^. A range of aquatic organisms including zooplankton^7^, benthic invertebrates^8^, bivalves^9^, fish^10^ and seabirds^11^ have the capacity to ingest microplastics, which may result in reduced feeding, energetic deficiencies, injury or death. Further, the large surface area to volume ratio and hydrophobic properties of microplastics make them prone to adhering waterborne contaminants, which may dissociate post-ingestion and cause toxicity^12^,^13^. The combined risks that microplastics pose to marine life are now widely recognized, and in recent years microplastic litter has been included in both national and international marine protection strategies, policies and legislature (e.g. EU Marine Strategy Framework Directive (MSFD), NOAA Marine Debris Program).

To monitor the spatial distribution and temporal trends of marine microplastic debris, it is vital that standardized protocols, capable of efficiently and accurately enumerating microplastics in a variety of habitats, are developed and implemented^14^. Methodologies suitable for detecting microplastic debris in both sediments and the water-column have been reviewed in detail by Hidalgo-Ruz *et al.*^15^. However, these sampling techniques will inevitably also collect biological material, sediment and detritus that can mask the presence of any microplastic litter present. With sediment samples, microplastic litter can be density-separated from surrounding silt and sediment using low-density salt (NaCl or NaI) solutions^16^; recent work has further suggested the use of fluvial elutriation chambers to increase separation efficiencies^17^. Plankton sampling has been highlighted as an effective method for collecting microplastic debris within the pelagic^18^,^19^. Samples taken from unproductive waters (e.g. oligotrophic oceanic gyres) typically contain low quantities of biological material, so identifying and isolating any microplastic debris can be done without the need for any separation techniques^20^,^21^,^22^,^23^. However, samples collected from highly productive waters (e.g. coastlines, convergence zones, etc.) can be full of plankton (Figure 1a), limiting our ability to observe and pick out any microplastics present. Developing methods that can be used to isolate microplastics from large volumes of biological material is currently a research priority^15^.

One of the main environmental risks associated with microplastics is their bioavailability to marine organisms. A number of studies have shown that microplastics can be ingested by marine biota under laboratory conditions; however, there is limited data relating to the biological uptake of microplastics *in-situ*. When studying larger field-collected organisms, including fish^10^ and crustaceans^24^, the intestinal tract can be dissected and any internalized microplastics enumerated. Van Franeker *et al.*^11^ have routinely applied this method with ocean-foraging Fulmars, providing important, novel data relating to microplastic concentrations at sea. However, dissection is less applicable for animals such as zooplankton and mussels, which can consume extremely small (2–31 μm diameter) microplastics^7^,^9^ that are not visible to the naked eye. Bio-imaging techniques including coherent anti-Stokes Raman scattering (CARS) microscopy^7^ have previously been employed to visualize the distribution of tiny microplastics internalized by zooplankton, but this method is time-intensive and can be hindered by algal pigments in natural specimens. Recently, Claessens *et al.*^17^ trialled the use of nitric acid to digest mussels to reveal ingested microplastics but found this caustic treatment destroyed some pH-sensitive polymers. As such, an alternate method is needed if a greater range of field-collected organisms are to be comprehensively bio-monitored for microplastic contamination.

In this study, we developed, optimized and validated a rapid and efficient protocol for digesting biological material without destroying microplastics. We compared the use of acid (HCl), alkaline (NaOH) and enzymatic (Proteinase-K) digestion treatments on plankton-rich seawater samples and a range of microplastics. We applied the optimized enzymatic protocol to replicate sub-surface seawater samples to isolate, enumerate and identify the microplastic debris present. We further used the enzymatic digestion protocol to reveal and quantify microplastics ingested by marine zooplankton.

## Results

### Mitigating contamination

Procedural blanks used during method development highlighted potential sources of contamination, including residue from aluminium foil lids used during the 60°C NaOH exposure, and plastic shavings following physical homogenization of samples within universal tubes. These sources of error were subsequently eliminated. Following method optimization, the microscopic analysis of filters retained from procedural blanks showed no evidence of microplastic contamination.

### Digestion efficacies

**Figure 1:**
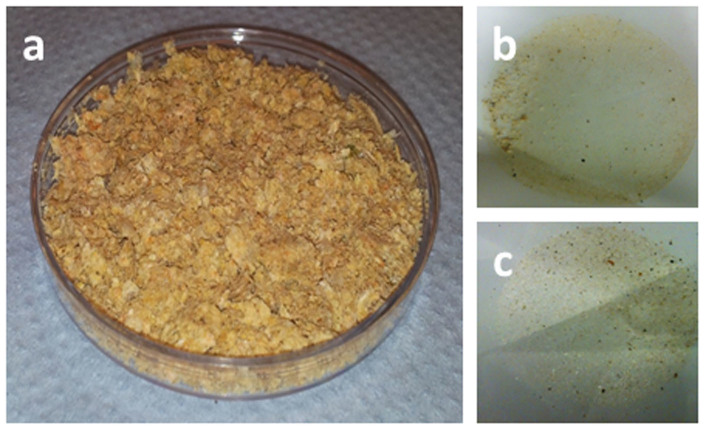
Digestion protocols can be applied to marine samples to remove biological material. (a) zooplankton collected from a 500 m sub-surface trawl following desiccation; the remnants of 0.2 g DW marine samples following (b) optimized alkaline and (c) optimized enzymatic digestion.

**Figure 2:**
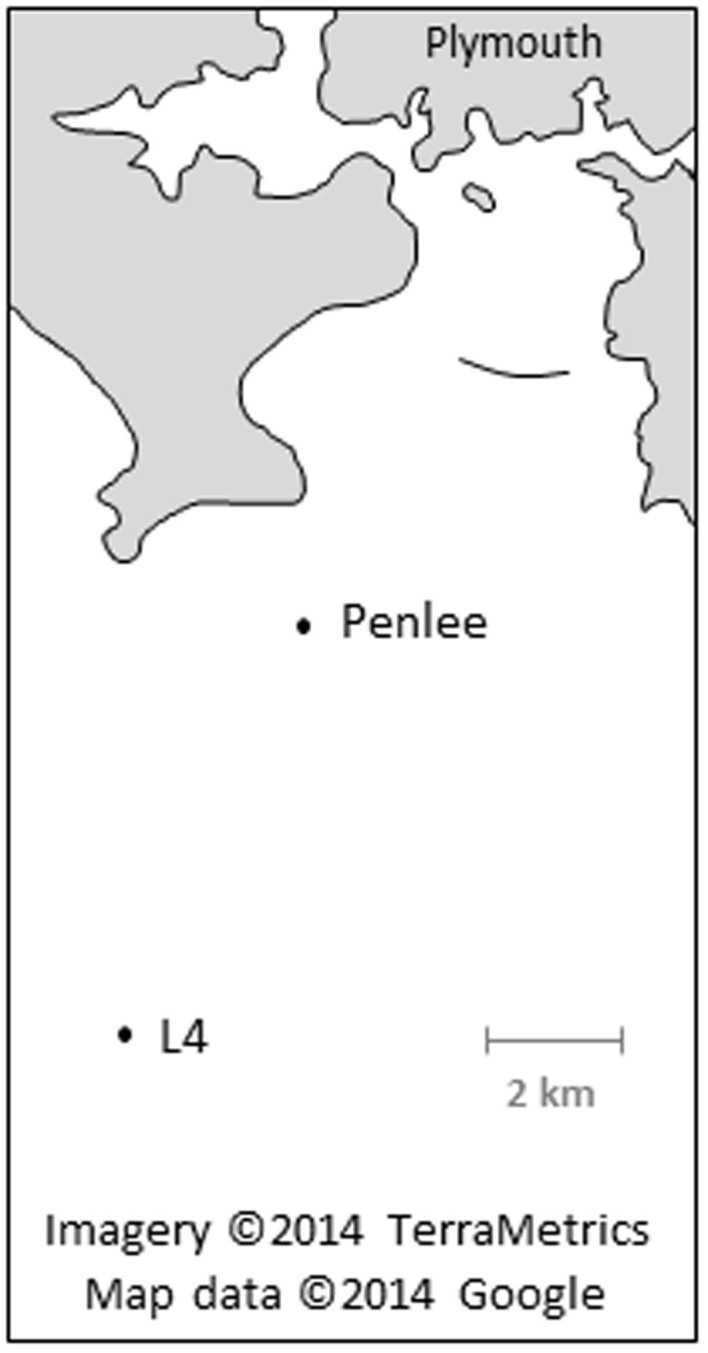
Map displaying the two marine sampling sites used in this study. Trawling was conducted in the western English Channel. Map created using a Google Maps overlay: Imagery ©2014 TerraMetrics; Map data ©2014 Google.

**Figure 3:**
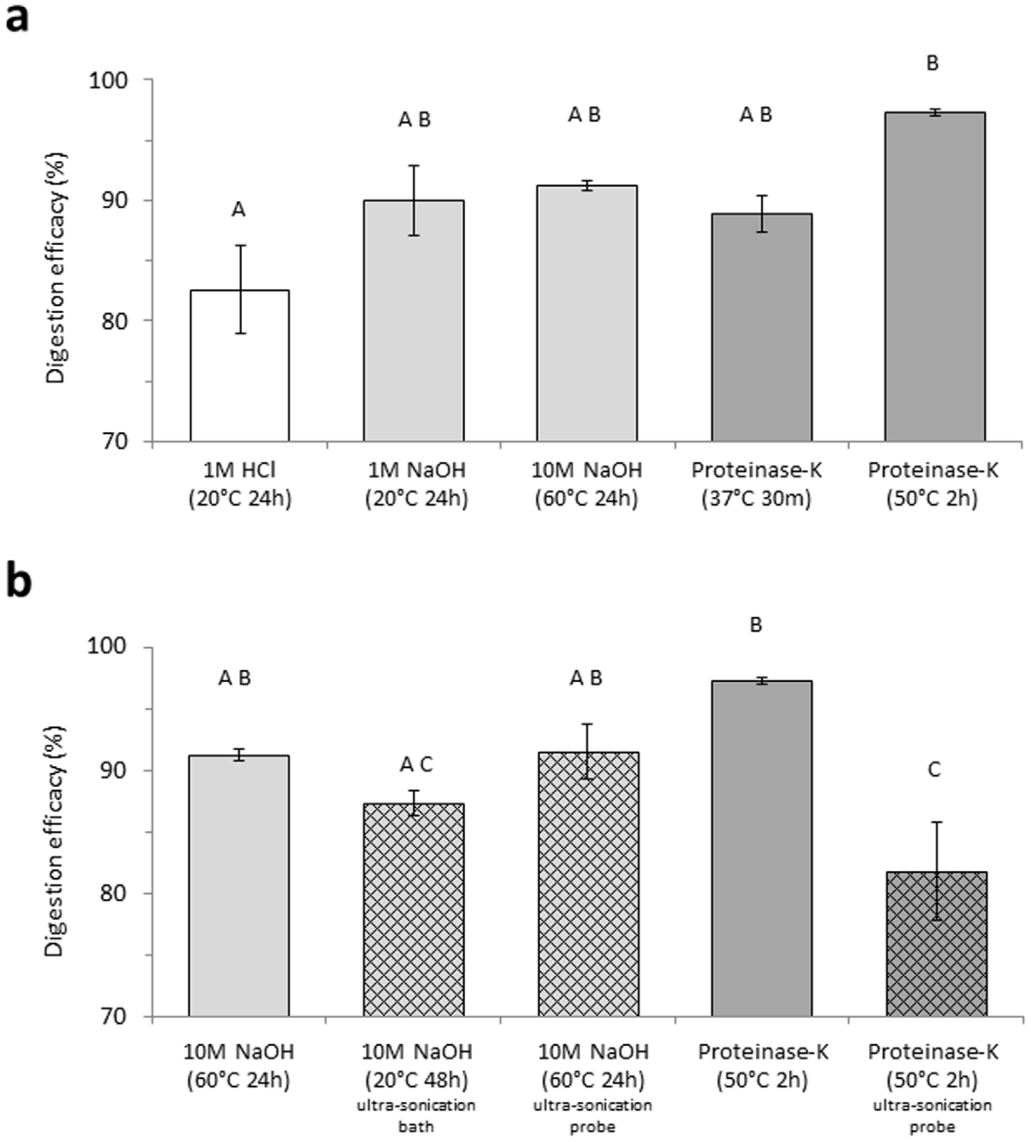
Digestion efficacy (%) – determined by weight – of acid/HCl [white], alkaline/NaOH [light grey] and enzymatic/Proteinase-K [dark grey] protocols applied to natural plankton samples. (a) comparison of initial and optimized digestion protocols; (b) comparison of optimized digestion protocols before and after ultrasonication [patterned].Data represents mean ± SEM (*n* = 3 per treatment; *n* = 4 with optimized Proteinase-K treatment). Letters denote significant difference between treatments (ANOVA with Tukey post-hoc analysis, *P* < 0.05).

The digestion efficacies of the treatments ranged from 54.0 to 97.7% (Figure 3). The least effective treatment was hydrochloric acid, with 1 M HCl (Figure 3a) and 2 M HCl (data not shown) resulting in digestion efficacies of 82.6 ± 3.7% and 72.1 ± 9.2% respectively. Comparatively, 1 M NaOH (Figure 3a) and 2 M NaOH (data not shown) digested the samples by 90.0 ± 2.9% and 85.0 ± 5.0% respectively. The optimized alkaline digestion protocol (10 M NaOH at 60°C) had a digestion efficacy of 91.3 ± 0.4% (Figure 1b; Figure 3a). Application of the original enzymatic protocol resulted in a digestion efficacy of 88.9 ± 1.5%, while, the optimized Proteinase-K treatment digested >97% of the samples and was significantly more effective than other treatments (Figure 1c; Figure 3a; ANOVA with Tukey post-hoc analysis, *P* < 0.05).

### Ultrasonication

Post-digestive ultrasonication was equally or less effective than using digestive treatments alone (Figure 3b). Use of an ultrasonication bath on 5 M and 10 M NaOH digested samples resulted in digestion efficacies of 90.9 ± 2.3% and 87.3 ± 1.0% respectively (Figure 3b). With samples digested using 10 M NaOH at 60°C, direct sonication increased digestion efficacy by just 0.3% (Figure 3b). Meanwhile, with enzymatically digested samples, direct sonication proved significantly less effective than using Proteinase-K alone (Figure 3b; t-test, *P* < 0.05).

### Filtering

**Figure 4:**
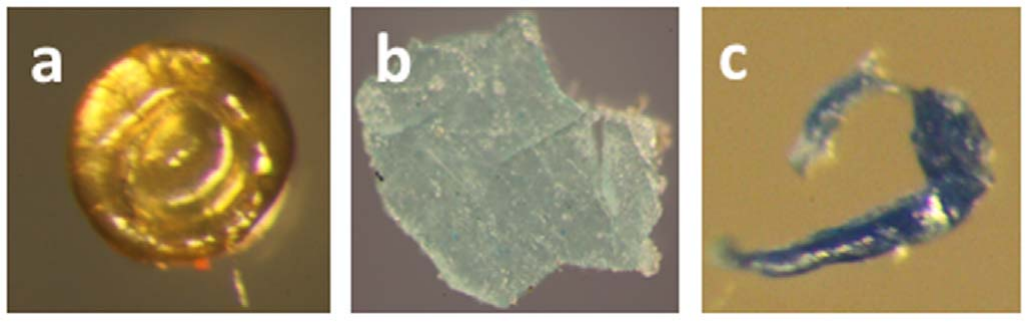
An array of microplastic litter – varying by shape, size, colour and polymer – was revealed by enzymatically digesting the biological material present within marine samples taken in the western English Channel. (a) a 140 μm diameter polyamide yellow-orange bead, (b) a 790 μm diameter grey-green polyethylene fragment, and (c) a 160 μm long blue uPVC fibre.

Visually, the optimized alkaline (Figure 1b) and enzymatic (Figure 1c) protocols were effective in reducing the biological material present within the marine samples. With both treatments, the undigested residue consisted of a thin, transparent film of glutinous biological material, in which microplastics (Figure 4) and small fragments of shell, wood and carapace were clearly visible. In comparing filters following optimized enzymatic digestion, we found 0.02 μm GF/Fs clogged quickly and were therefore inappropriate for this task; while 50 μm mesh-filters had the fastest filtration rates, 20 μm mesh-filters will theoretically capture smaller microplastics without significantly impacting upon digestion efficacy (50 μm filter: 97.3%; 20 μm filter: 97.1%; t-test, *P* = 0.67).

### Impact of optimized digestion protocols on microplastics

**Figure 5:**
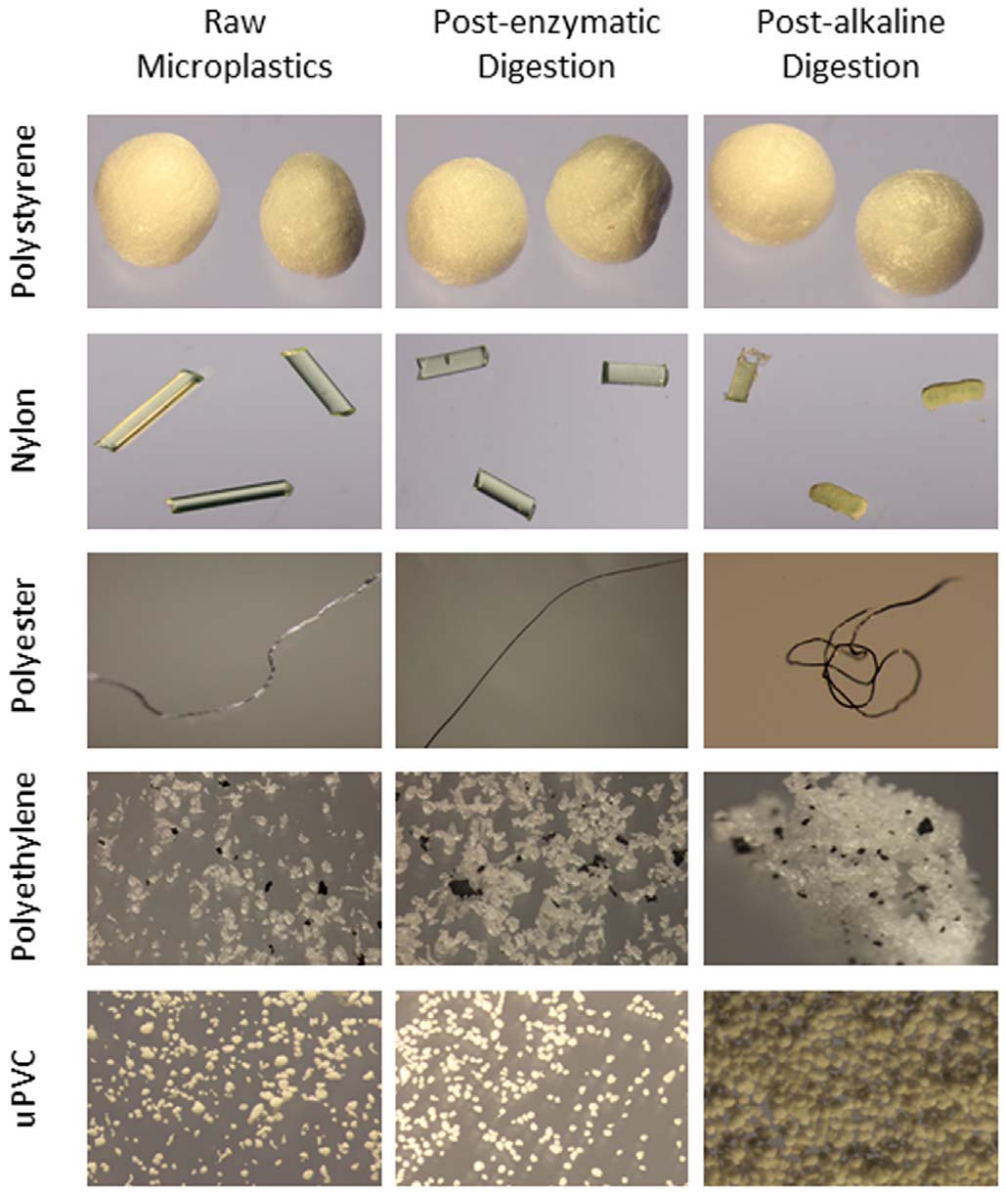
Microplastics photographed before and after enzymatic and alkaline digestion. Alkaline hydrolysis resulted in structural damage to Nylon fibres, melding of polyethylene fragments and discoloration to uPVC granules. Magnification: polystyrene spheres x40; Nylon line x20; polyester fibre x63; polyethylene granules x200; uPVC powder x160.

The optimized enzymatic protocol showed no visible impact on any of the microplastics undergoing treatment (Figure 5). Conversely, the optimized alkaline treatment resulted in the partial destruction of Nylon fibres, melding of polyethylene fragments, and a yellowing of uPVC granules (Figure 5); further, several polyester fibres were lost in applying this protocol (data not shown).

### Validation of the enzymatic digestion with *in-situ* microplastic debris

**Figure 6:**
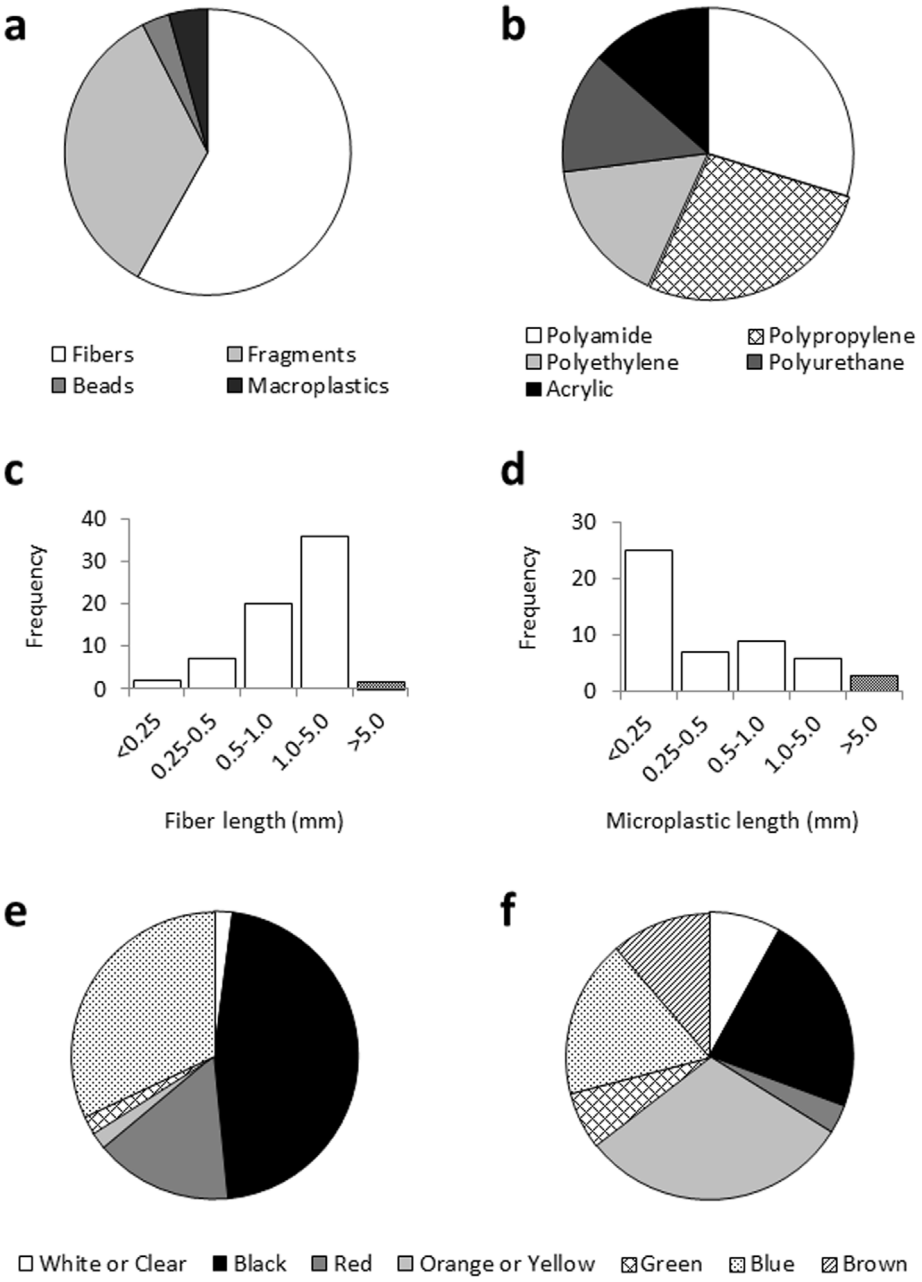
Plastic litter extracted from two western English Channel sites varied by form, polymer, size and colour. (a) suspect debris was categorized as microplastic fibre, fragment or bead, or macroplastic (>5 mm) (*n* = 162); (b) FT-IR analysis was used to determine the constituent polymer of a sub-sample (*n* = 40) of the suspected microplastic litter (note: 3 of these items were categorized as non-plastic, excluded from figure); sizing was conducted by either measuring (c) the length of a sub-sample of fibres (*n* = 67), or (d) the widest diameter of a sub-sample of non-fibrous plastic litter present (*n* = 50); colour was categorized for both (e) fibrous (*n* = 97), and (f) non-fibrous litter (*n* = 62) present.

In all, 162 suspected plastics were isolated in four trawl samples taken in the western English Channel, of which 96% were classified as ‘microplastic' (Figure 6). Concentrations of suspected microplastics averaged 0.26 items m^−3^ at Penlee, and 0.31 items m^−3^ at L4 (SI Table 2). FT-IR analysis revealed the majority of this material was plastic, primarily consisting of polyamide (e.g. Nylon) or polypropylene (Figure 6b). Notably, two of these 40 (5%) items were identified as leather (data not shown). The majority of the isolated micro-debris was fibrous (61%; Figure 6a), with widths varying between 6 and 175 μm and lengths >250 μm (Figure 6c). Non-fibrous microplastics, including granular or planar fragments (36%) and beads (3%), were predominantly <250 μm in diameter (Figure 6d). Fibres were most commonly found to be black, blue or red (Figure 6e), while non-fibrous plastics had a more even spread of colours (Figure 6f).

### Detecting microplastics ingested by copepods

**Figure 7:**
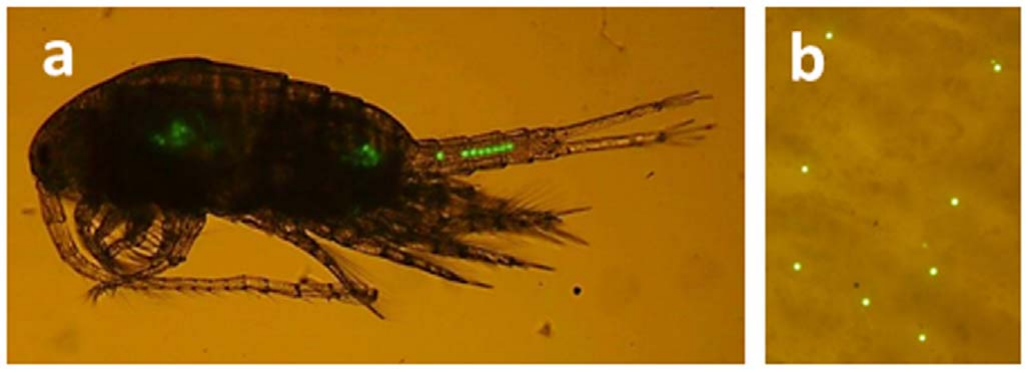
Microplastics ingested by zooplankton can be quantified after enzymatic digestion of the specimens. (a) 20 μm diameter fluorescent polystyrene microplastics internalized by the pigmented calanoid copepod *Temora longicornis*; (b) following enzymatic digestion the biological tissue was mineralized, releasing the fluorescent microplastics, which were then retained on a glass fibre filter.

Microplastics were ingested by 11 of the 18 (77%) *T. longicornis*; fluorescent beads were clearly visible in the hindgut, but obscured by the opaque, pigmented carapace when in the fore-gut (Figure 7a). The enzymatic protocol digested the copepod tissue and the microplastics were retained on a GF/F (Figure 7b). With fluorescence enabled, microplastics were successfully enumerated, indicating an average load of 10.7 ± 2.5 beads per copepod (at the point at which the experiment was terminated) (SI Table 3).

## Discussion

Here, we have optimized an enzymatic digestion protocol capable of mineralizing marine zooplankton without causing damage to microplastics. This rapid and efficient method proved capable of digesting >97% of the material present within sub-surface marine samples, allowing the microplastic litter present to be isolated with ease. The protocol showed low risk of external contamination and was proven applicable to entire trawl samples taken from a site with high biological productivity. Furthermore, the protocol proved successful in releasing microplastics ingested by zooplankton, highlighting the method's potential application for detecting whether marine organisms are ingesting microplastic debris in the wild.

The ubiquity of microplastics in sea-surface, water-column and sediment samples from across the globe has highlighted the prevalence of this contaminant within our oceans^3^,^28^. Microplastics can be ingested by a range of marine biota, including shellfish and fish fit for human consumption^9^,^10^, and can result in adverse health impacts to these organisms^4^. As such, national, regional and global bodies now widely consider microplastics to be a marine pollutant. In the EU, the MSFD (Descriptor 10.1.3: Marine Litter) requires that trends in microplastic abundance and distribution be monitored within European waters^29^. While microplastic pollution in oceanic gyres^20^,^21^,^22^,^23^ and coastal sediments^28^,^30^ has been well reported in recent years, there has been limited focus on microplastics in near-shore waters. Coastlines are prone to fresh inputs of anthropogenic litter from run-off, urban waterways and sewage outfall^31^,^32^ but are also highly productive marine habitats. As microplastics pose a major threat to marine biota, providing data on bioavailable microplastics in coastal waters is of growing importance.

Traditionally, acid digestion might be used to remove biological material, such as plankton, present in marine samples (e.g. for analysis of trace metals); this method typically uses strong mineral acids (e.g. H_2_SO_4_, HNO_3_), either in open or closed systems in conjunction with high temperature and pressure, which can oxidize compounds, causing molecular cleavage^33^. However, these oxidizing acids can also destroy or damage polymers with a low pH tolerance (e.g. polyamide, polystyrene)^17^, thus the application of acid digestion in the analysis of microplastics is limited. As an alternative, we used low concentrations of the non-oxidizing, mineral acid HCl at room temperature. In digesting plankton, HCl proved inconsistent and inefficient, with large quantities of material remaining on the filters post-digestion. A viable alternative is alkaline hydrolysis, in which strong bases (e.g. NaOH, KOH) are used to denature proteins and hydrolyse compounds^34^. Use of 1 M NaOH at room-temperature proved 90% effective in digesting 0.2 g DW marine samples; by increasing molarity and experimental temperature, digestion efficacies were both increased and stabilized. The procedural blanks used with this protocol showed that aluminium foil lids (used to prevent airborne contamination) were degenerating from the high pH with higher temperature incubations, and as such were soiling the samples; this issue was rectified by replacing the aluminium lids with glass caps. Recently Nuelle, *et al*.^16^ have demonstrated that hydrogen peroxide (H_2_O_2_) is a more effective agent for removing biogenic material from sediment samples than HCl or NaOH. However, use of 35% H_2_O_2_ over a relatively long exposure period of 7 days resulted in the complete removal of just 25% of the biological material (<1 mm) present. Remaining biogenic material was bleached, an issue which the authors note may interfere with the isolation of microplastics from the samples.

Ultrasonication can be used to disintegrate, fragment or solubilize solutes by using high-frequency sound waves to cavitate media, creating high sheer forces^21^. In the preparation of sewage sludge, Jin *et al.*^21^ found that using a combination of alkaline hydrolysis and ultrasonication increased digestion efficacy by an average of 7.7% compared with using alkaline hydrolysis or ultrasonication alone. However, our data showed that neither the use of an ultrasonication bath or probe was any more effective than using NaOH alone. While the optimized alkaline digestion method was suitable for digesting plankton, it also proved damaging to microplastics: exposed to 10 M NaOH at 60°C, three of the five polymers (Nylon, polyethylene and uPVC) were damaged or discoloured. When applied to marine samples, use of alkaline hydrolysis could therefore result in an under-representation of pH-sensitive polymers or miscataloging of microplastic by colour, and as such cannot be recommended for this purpose. Similarly, while many plastics are resistant to hydrogen peroxide, Nuelle, *et al*.^16^ recently demonstrated that 35% H_2_O_2_ can result in significant (15.9 – 17.2%) size losses in exposed polyethylene and polypropylene microplastics.

The proteolytic enzyme Proteinase-K has previously been used to extract DNA from zooplankton specimens prior to molecular analysis^25^. In using this protocol we attained a digestion efficacy of 88%, which was increased to >97% by increasing the concentration of Proteinase-K used, raising the active temperature to 50°C and prolonging the incubation period. Use of procedural blanks highlighted that by physically homogenizing our samples within plastic universal tubes we were introducing plastic shavings to the sample, which was rectified by switching to acid-washed glass containers. Undigested material consisted of fragments of shell, carapace, wood and anthropogenic litter embedded within a thin film of a clear, glutinous biological material. Although this biological material was not further analysed, we hypothesize it consisted of chitin, an insoluble, polysaccharide-based biopolymer present in zooplankton carapaces^35^. Microplastic litter within the samples could be identified and extracted from this film without issue. However, if this chitin were problematic in future applications of this protocol then the enzyme chitinase could be applied to break down this residue. Bermejo *et al.*^36^ demonstrated that proportionate ultrasonication increases the specific activity of enzymes in solution. However, in ultrasonicating our enzymatically digested marine samples, we noted a substantial decrease in digestion efficacy compared with using enzymatic digestion alone. This likely resulted from the formation of protein precipitates within the digestion media, formed as a by-product of intense cavitation^37^, which were then unable to pass through the mesh-filters post-treatment. In contrast with the acid and alkaline digestion, enzymatic digestion is conducted at a neutral pH and moderate temperature and has biological specificity^36^. It was therefore of little surprise that the enzymatic protocol had no discernible effect on any of the five microplastics exposed. We would therefore consider enzymatic digestion to be highly applicable in removing biological material from biota-rich marine samples to elucidate microplastic pollution.

The optimized enzymatic protocol was trialled on whole-trawl samples collected from the western English Channel, and revealed 0.30 items of anthropogenic debris m^−3^. FT-IR analysis revealed that while the majority of suspected plastics consisted of a synthetic polymer, ~5% of these items were made of leather. In extrapolating this data, and by defining a microplastic as <5 mm diameter, we identified an average of 0.27 items of microplastics debris m^−3^ in the western English Channel trawls. This value is consistent with the broad range of concentrations (0.014–12.51 items m^−3^) observed in similar studies conducted across the globe^15^. By using finer, 200 μm nets in combination with enzymatic digestion, we successfully identified 27 fibres, fragments and beads 25–250 μm in size across our four sub-surface trawls. Data relating to microplastics within this size range is scant. Only two of 33 studies investigating pelagic microplastics used nets finer than 300 μm aperture^15^. Yet it is microplastics of this size that can be ingested by zooplankton and benthic invertebrates, resulting in reduced feeding rates and declining energetic reserves^7^,^8^. Lusher *et al.*^10^ recently identified that 36.5% of 544 pelagic fish sampled at study site L4 in the English channel (described above) contained microplastics (comparable in size to those found within our samples) within their intestinal tracts. Within our samples, polyamides (e.g. Nylon) and polyethylene were some of the most commonly noted polymers; these pH-sensitive polymers may have been destroyed if acid^17^, alkaline or hydrogen peroxide^16^ were used on the samples, whereas they survived enzymatic digestion. It would be presumptuous to draw further conclusions from these trial samples alone. Nevertheless, based on our findings we can recommend enzymatic protocols to detect microplastics in plankton samples in future studies.

The smallest microplastics identified in our sample were brightly coloured fragments 25 μm in diameter. Microplastics of this size may be retained on wider aperture mesh-filters and nets when they become clogged, although it is also possible that plastic litter of this size might have been internalized by zooplankton present within the sample. However, we cannot rule out the possibility that desiccating and grinding samples using a mortar and pestle may result in thermal and abrasive degradation of larger microplastics within the sample. This concern could be mitigated by using alternate preservation techniques (e.g. formalin, ethanol) and avoiding physical grinding, however, the efficacy of enzymatic digestion may be diminished where preservatives have been applied and the available surface area of the biological material has not been optimised. The range of colours identified suggest bleaching of plastics was not an issue, and stands as further proof that enzymatic digestion has minimal effects on plastics. Despite the commonality of white or clear plastics used in packaging, fishing line, clothing, personal care products and other plastic products, we did not isolate any uncoloured microplastics <250 μm in our samples. Furthermore, in considering shape, the majority of plastic litter identified in our sample (and in sediment and water-column samples from around the world^15^) were fibrous. While such trends may be representative of the microplastics present in the marine environment, it is also important to consider that there may be a strong operator selection bias towards fibrous and brightly coloured microplastics owing to the relative ease of their identification.

The enzymatic digestion protocol was also successfully used to reveal fluorescent polystyrene beads ingested by a marine copepod. Previously, Claessens *et al.*^17^ trialled using nitric acid digestion to detect microplastics ingested by mussels, but found that both Nylon and polyethylene could not survive this harsh, oxidizing treatment. As enzymatic digestion is biologically specific, it is able to digest tissue without damaging plastics, and therefore proved highly applicable in this study. Fluorescent beads retained on the filter were enumerated with ease. While we anticipate that this protocol would be ideal for digesting field-collected zooplankton (and perhaps larger marine organisms) to look for evidence of microplastic ingestion, better techniques for visualizing and/or identifying very small non-fluorescent microplastics are still required.

## Conclusion

We have developed a rapid method in which a proteolytic enzyme treatment can be applied to marine samples to digest away biological material without damaging any microplastics present. As research shifts from oligotrophic gyres to biologically rich coastal systems, it is hoped that this enzymatic protocol can be widely applied to better identify microplastics within these vulnerable ecosystems. By using this method in conjunction with finer sampling nets, data gaps relating to the concentration of microplastics <300 μm can be addressed. The enzymatic protocol has also been shown as an apt technique for identifying microplastics internalized by marine zooplankton. Further work is now required to trial the technique on field-collected specimens and to develop means of better visualizing very small, ingested microplastics.

## Methods

### Zooplankton sampling

Development of the digestion technique was performed on zooplankton collected as part of the long-term time series at a research station located in the English Channel (station L4), located 12 km south of Plymouth, UK (50°15′N, 04°13′W; Figure 2; www.westernchannelobservatory.org.uk). Horizontal, sub-surface tows using 200 μm and 500 μm plankton nets were used to collect zooplankton throughout August and September 2013. These biota-rich samples were transferred to clean, insulated containers, and transported to Plymouth Marine Laboratory (PML) within two hours of trawling.

### Sample preparation

Zooplankton samples were passed through 200 μm meshes, and retained material was flushed with purified water (Milli-Q) to remove salt. Any macrozooplankton or large items of debris were rinsed thoroughly and then removed from the sample. Meshes were folded and sealed, and then oven-dried at 60°C for >24 hours to both preserve samples and enhance the efficacy of grinding up the biological material in later steps. Desiccated samples were carefully ground with a mortar and pestle to increase the surface area of the biological material present. For method development and optimization, desiccated samples were pooled and 0.20 g DW sub-samples weighed out and then carefully transferred into digestion vessels.

### Mitigating contamination

As microplastics may be airborne, present in clothing or adhered to laboratory equipment, we undertook a number of steps to prevent sample contamination. All apparatus were acid-washed and/or rinsed thoroughly with Milli-Q prior to use, while consumables were used directly from packaging. Sample preparation was conducted within a sterile algal-culturing unit. Samples and equipment were covered wherever possible to minimize periods of exposure. Personal protective equipment was worn at all times. Procedural blanks (absent of biological material or microplastics) were run in parallel with samples containing dessicated plankton, microplastics or trawled material. To demonstrate the efficacy of our preventive measures, procedural blanks were poured through mesh filters (per the methods below) and retained material analysed under a digital light microscope (Olympus SZX16) to check for contamination.

### Acid and alkaline digestion

To compare the efficacy of acid or alkaline hydrolysis in digesting marine zooplankton, we used hydrochloric acid (HCl) or sodium hydroxide (NaOH) treatments. In brief: 20 mL of 1 M HCl, 2 M HCl, 1 M NaOH or 2 M NaOH (*n* = 3 per treatment) were added to glass flasks containing the sub-samples and maintained at room temperature for 24 h. We further trialled 5 M and 10 M NaOH at room temperature for 48 h, and 1 M and 10 M NaOH at 60°C for 24 h (*n* = 3 per treatment). The optimized alkaline protocol required 40 mL of 10 M NaOH per 0.2 g dry weight (DW) sample, maintained at 60°C for 24 h. Prior to analysis, samples were neutralized using HCl or NaOH as required.

### Enzymatic digestion

The enzymatic digestion protocol developed by Lindeque and Smerdon^25^, was adapted to use 500 μg mL^−1^ of Proteinase-K per 0.2 g DW sample, within universal tubes (*n* = 3). To maximize digestion efficacy and avoid contamination, desiccated samples were transferred into 50 mL acid-washed, screw-top glass containers with 15 mL homogenizing solution (400 mM Tris-HCl buffer, 60 mM EDTA, 105 mM NaCl, 1% SDS). Samples were physically homogenized by drawing and expelling the mixture through a 19G needle attached to a 10 mL syringe, making sure to thoroughly rinse the insides of the needle and syringe with homogenizing solution to avoid loss of material. Samples were incubated at 50°C for 15 minutes, and then 500 μg mL^−1^ of Proteinase-K added before incubating the samples at 50°C for a further 2 hours. Next, 5 M sodium perchlorate (NaClO_4_) was added and samples shaken at room temperature for >20 minutes. Solutions were physically homogenized a second time using a finer 21G needle, and then incubated at 60°C for 20 minutes.

### Ultrasonication

We employed two ultrasonication techniques to assist in the breakdown of digested samples: (a) the 5 M and 10 M NaOH digestions were placed in an ultrasonication bath for 10 minutes; (b) a sonication probe was used to directly sonicate samples that had undergone alkaline (1 M NaOH, 60°C and 10 M NaOH, 60°C) and enzymatic (Proteinase-K, 50°C, 2 h) digestion. With both methods, samples were ultrasonicated on ice to prevent excess heat.

### Digestion efficacies

Post-digestion (and ultrasonication, where applicable) samples were vacuum-filtered onto pre-weighed 50 μm mesh-filters. Retained biological material was flushed copiously with Milli-Q, and then filters removed, covered and oven-dried at 60°C. Following desiccation, filters were weighed and digestion efficacies calculated by comparing the relative removal of organic mass during the digestion. In a follow-up experiment, we compared the use of 50 μm, 20 μm and 0.02 μm filters (*n* = 3 per filter size) following a series of optimized enzymatic digestions.

### Microplastics

Five types of microplastic, varying by polymer, shape, size and colour and representative of the array of microscopic plastic debris present in marine ecosystems were sourced for method validation: (a) white expanded polystyrene spheres (1.5–3.0 mm in diameter) were accrued from packaging material; (b) yellow-green Nylon monofilament fishing line (400 μm diameter) was cut into ~1 mm lengths; (c) black polyester fibres (10–30 μm diameter, with lengths of 0.75–1.5 mm) were individually plucked from a 100% polyester garment; (d) 100 mL of a domestic personal care product was mixed with 1 L of Milli-Q, and then filtered through a 50 μm mesh to retain a combination of black and white polyethylene fragments (60–500 μm diameter); (e) lastly, we purchased white unplasticized polyvinylchloride (uPVC) granules (60–120 μm diameter; Goodfellows). Microplastics were measured using a microscope (Olympus SZX16; x16–62 magnifcation) fitted with an eye-piece graticule or *cellSens* software (Olympus). The constituent polymers of the microplastics were confirmed using Fourier transform infrared spectroscopy (FT-IR; Bruker Hyperion FT-IR microscope) in conjunction with a reference database^3^.

### Impact of optimized digestion protocols on microplastics

Optimized alkaline and enzymatic digestion protocols were applied directly to each of the five selected microplastics. Following the prescribed method, microplastics were retained on 50 μm mesh-filters, rinsed with Milli-Q and then oven-dried. Microplastics were weighed (uPVC granules, polyethylene fragments) or counted (polystyrene spheres, Nylon fibres, polyester fibres), and subsequently checked for damage under a microscope.

### Validation of the enzymatic digestion with *in-situ* microplastic debris

We applied the optimized enzymatic digestion method to replicate trawl samples from two sites in the western English Channel (Figure 2). Sampling was conducted in mid-October 2013 using two 200 μm-aperture WP2 nets towed in parallel for approximately 500 m (SI Table 1). The volume of water filtered (V) was calculated by considering the radius of the net aperture (r) and length of tow (L) as confirmed via GPS, and applying a 95% filtering efficiency specific to 200 μm WP2 nets^26^: V = (πr^2^) × L × 0.95. Samples were transported, filtered, rinsed, desiccated and ground as previously described (see *Sample Preparation*), without pooling material, and then the optimized enzymatic digestion protocol applied to each sample. To compensate for the greater amount of biological material undergoing digestion (0.60–0.96 g DW), the volume of homogenizing solution, Proteinase-K and sodium perchlorate was increased. Further, the undigested material present in each sample was split between three 50 μm mesh-filters to prevent clogging and to more easily identify any microplastics present. Filters were analysed under a digital light microscope (Olympus SZX16) and suspected microplastics (assessed by colour, uniformity of material and shape^27^) were isolated, classified, measured and subsequently analysed using FT-IR to determine their polymer.

### Detecting microplastics ingested by copepods

The optimized enzymatic digestion protocol was further considered as a technique for detecting microplastics ingested by marine organisms. Specimens of the marine copepod *Temora longicornis* were isolated from a zooplankton trawl, and then three individuals (*n* = 5) placed in a Petri-dish containing 20 mL of filtered seawater containing fluorescent polystyrene beads (100 microplastics mL^−1^) overnight at ambient sea surface temperature. Post-exposure, specimens were retained on a mesh-filter, preserved using 4% formalin and rinsed thoroughly with Milli-Q. Copepods were visualized under a microscope (fitted with fluorescence) to quantify the number of specimens that had ingested the polystyrene beads and it was confirmed that no external microplastics were present. *T. longicornis* were enzymatically digested per the standardized protocol, using smaller volumes of homogenizing solution, Proteinase-K and sodium perchlorate owing to the smaller mass of biological material being digested. Digested extract was filtered onto a 0.2 μm glass fibre filter (GF/F) and residue visualized under a microscope to enumerate and photograph the microplastics that had been previously internalized by the copepods.

### Statistics

Data is presented as mean ± SEM. A student's t-test (Microsoft Excel: two-tailed t-test) and one-way ANOVA with Tukey post-hoc analysis (Minitab) were used to compare the digestion efficacies of different treatments, with statistical significance attributed where *P* < 0.05.

## Author Contributions

M.C. conceived and designed the experiments. M.C. and H.W. conducted the experimental work and analysed the data. M.C. wrote the paper. P.L., E.F., C.H. and T.G. provided their expertise and reviewed the manuscript.

## Supplementary Material

Supplementary Information Supplementary Information
