# Supplementary material for: Isolation of microplastics in biota-rich seawater samples and marine organisms
Source: Sci Rep. 2014 Mar 31;4:4528. doi: 10.1038/srep04528 (PMC3970126; doi:10.1038/srep04528)
Supplement: Supplementary Information [file srep04528-s1.doc]

**Isolation of microplastics in biota-rich seawater samples and marine organisms**

Matthew Cole^a,b,*^, Hannah Webb^c^, Pennie Lindeque^a^, Elaine Fileman^a^, Claudia Halsband^d^ & Tamara S. Galloway^b^

**SI Table 1: Sampling conditions for western English Channel.**

| *Site* | *Date* | *Time* | *Start*  *[Latitude Longitude]* | *Finish*  *[Latitude Longitude]* | *Distance*  *[km]* | *Beaufort*  *scale* | *Swell*  *[ft]* | *Wind spd.*  *[knots]* | *Wind direction* | *Tide*  *[Ebb/Flood]* | *Tide*  *[Low/High]* |
| --- | --- | --- | --- | --- | --- | --- | --- | --- | --- | --- | --- |
| Penlee | 14.10.13 | 11:00 | 50°17’90”N 04°11’32”W | 50°18’24”N 04°11’15”W | 0.66 | 2 | 1–2 | 13 | NW | Flood | Low |
| L4 | 14.10.13 | 10:30 | 50°14’99”N 04°13’17”W | 50°15’20”N 04°13’41”W | 0.48 | 2 | 1–2 | 13 | NW | Flood | Low |

**SI Table 2: Debris identified from replicate 500 m horizontal trawls, using 200 µm plankton nets, at two sites in the western English Channel.**

|  |  | *Microplastic* | | | | *Macroplastics  (>5 mm)* | *Volume filtered (m^3^)* | *Debris concentration (items m^-3^)* | *Microplastic concentration(items m^-3^)** |
| --- | --- | --- | --- | --- | --- | --- | --- | --- | --- |
|  |  | *Beads* | *Fragments* | *Fibers* | *Total* |  |  |  |  |
| Penlee | Trawl A | 1 | 12 | 25 | 40 | 3 | 160.0 | 0.27 | 0.24 |
|  | Trawl B | 0 | 18 | 25 | 43 | 0 |  | 0.27 | 0.26 |
| L4 | Trawl A | 0 | 14 | 17 | 31 | 4 | 116.4 | 0.30 | 0.25 |
|  | Trawl B | 4 | 11 | 26 | 41 | 0 |  | 0.35 | 0.33 |
| Control | Blank A | 0 | 0 | 0 | 0 | 0 | N/A | N/A | N/A |
|  | Blank B | 0 | 0 | 0 | 0 | 0 |  |  |  |

* assuming 95% of suspected microplastics are synthetic polymers

**SI Table 3: Microplastic ingestion by the copepod Temora longicornis.**

| Rep: | A | B | C | D | E |
| --- | --- | --- | --- | --- | --- |
| Number of individuals (maximum: 3) showing microplastic ingestion | 3 | 2 | 2 | 1 | 3 |
| Number of microplastics identified following enzymatic digestion | 44 | 20 | 48 | 9 | 39 |
| Average number of microplastics ingested per copepod | 14.7 | 6.7 | 16.0 | 3.0 | 13.0 |
